# Construct Validity of Functional Capacity Evaluation in Patients with Whiplash-Associated Disorders

**DOI:** 10.1007/s10926-014-9555-0

**Published:** 2014-11-11

**Authors:** M. A. Trippolini, P. U. Dijkstra, J. H. B. Geertzen, M. F. Reneman

**Affiliations:** 1 Department of Work Rehabilitation, Rehaklinik Bellikon, Suva Care, 5454 Bellikon, Switzerland; 2 Department of Rehabilitation Medicine, Center for Rehabilitation, University Medical Center Groningen, University of Groningen, Groningen, The Netherlands; 3 Department of Oral and Maxillofacial Surgery, University Medical Center Groningen, University of Groningen, Groningen, The Netherlands

**Keywords:** Whiplash injuries, Neck pain, Disability evaluation, Lifting, Sick leave, Population groups

## Abstract

*Purpose* The construct validity of functional capacity evaluations (FCE) in whiplash-associated disorders (WAD) is unknown. The aim of this study was to analyse the validity of FCE in patients with WAD with cultural differences within a workers’ compensation setting. *Methods* 314 participants (42 % females, mean age 36.7 years) with WAD (grade I and II) were referred for an interdisciplinary assessment that included FCE tests. Four FCE tests (hand grip strength, lifting waist to overhead, overhead working, and repetitive reaching) and a number of concurrent variables such as self-reported pain, capacity, disability, and psychological distress were measured. To test construct validity, 29 a priori formulated hypotheses were tested, 4 related to gender differences, 20 related associations with other constructs, 5 related to cultural differences. *Results* Men had significantly more hand grip strength (+17.5 kg) and lifted more weight (+3.7 kg): two out of four gender-related hypotheses were confirmed. Correlation between FCE and pain ranged from −0.39 to 0.31; FCE and self-reported capacity from −0.42 to 0.61; FCE and disability from −0.45 to 0.34; FCE and anxiety from −0.36 to 0.27; and FCE and depression from −0.41 to 0.34: 16 of 20 hypotheses regarding FCE and other constructs were confirmed. FCE test results between the cultural groups differed significantly (4 hypotheses confirmed) and effect size (ES) between correlations were small (1 hypothesis confirmed). In total 23 out of 29 hypotheses were confirmed (79 %). *Conclusions* The construct validity for testing functional capacity was confirmed for the majority of FCE tests in patients with WAD with cultural differences and in a workers’ compensation setting. Additional validation studies in other settings are needed for verification.

## Introduction

The term whiplash-associated disorders (WAD) has been coined for symptoms related to acceleration-deceleration injuries usually associated with motor vehicle accidents [1]. These symptoms include neck pain, headache, arm pain, and other complaints [1]. The aetiology of WAD likely combines physical and psychological factors; nevertheless, the pathophysiology is not understood [2]. Although the prognosis of WAD is generally favourable, with a recovery rate of 40–60 % within the first 12 months, a considerable number of individuals with WAD still reports symptoms and disability, 1 year after the injury [3, 4]. Delayed recovery of WAD causes a substantial burden for the individual and society due to long-term sickness, absence, and work disability [5].

According to the guidelines of the International Labor Organization, diseased or disabled persons should be assessed comprehensively to avoid an over- or underestimation of safe work (dis)ability [6]. Functional capacity evaluation (FCE) can be one of the tools included in such an assessment. FCE consists of standardised batteries of functional capacity tests that aim to measure the ability to engage in work-related functioning [7]. When discrepancies between FCE outcomes and the physical workload indicate that capacity is not large enough for the required work load, this capacity may be addressed in rehabilitation programmes to reduce these discrepancies [8, 9]. Moreover, FCEs are used to determine fitness-for-work, and may facilitate the return-to-work process or prelude case closure [10, 11].

Functional capacity (FC) has been defined as the highest probable level of function that a person may reach in a domain at a given moment in a standardised environment [8]. Functional capacity is a multidimensional, bio-psycho-social construct, which means that FC is the result of biological and psychological abilities, positively or negatively influenced by personal and external (social) factors (e.g., test environment, education, family) [8, 9]. No gold standard exists for the measurement of FC, therefore, validity must be determined by means of construct validity. Construct validity is the degree to which a particular measure relates to other measures in a way one would expect, i.e., in accordance with predefined hypotheses about the correlation or differences between the measures [10]. From a biological perspective, within the bio-psychosocial construct of FC, it can be expected that males are stronger than females and score higher on material handling and grip strength tests, and score similar in postural tolerance and repetitive work tests [11, 12]. From a psychological viewpoint it can be hypothesized that in patients with WAD, FC correlates with self-reported pain and mental distress to a larger extend than in healthy workers [4, 13]. However, the correlation between FC and mental distress is expected to be smaller compared to the correlation between FCE tests and other measures of functional ability and disability [9, 14]. Additionally, the socio-cultural context may influence FC due to different cultural representations and expectations [15]. A study comparing FCE test results of patients with chronic low back pain (CLBP) in three different countries showed substantial differences between the study samples [16]. People from different ethnic backgrounds living in the same country reported musculoskeletal pain differently [17–19]. One can assume that FCE tests may result in differences between groups with different cultural backgrounds. However, this has not yet been studied.

For both, clinician and researcher it is important to know, how other measures are related to FC, in order to understand what is measured by FCE tests. Because clinical decision-making is based on the results of FCE tests, sound clinimetric properties of FCE tests are required [20]. During the past decades, reliability and, to a lesser extent, validity and safety of FCEs have been studied predominantly in patients with CLBP [10, 21] and in one study in healthy persons [13]. FCE validity research should also be conducted in other chronic health conditions such as patients suffering from WAD, because clinimetric properties may not be generalisable across health conditions [22] and cultural settings [23]. Many studies on the construct validity of FCE tests did not meet the requested quality criteria such as formulating an a priori hypothesis for the strength of correlation and adequate sample size [9]. Moreover, few FCE tests were able to demonstrate adequate validity in more than one study and more than one health condition area [24].

Hence, the aim of this study was to analyse the construct validity of the FCE test for a large sample of patients with WAD, from various cultural backgrounds, who did not return to work after injury onset and who received workers’ compensation, using a priori defined hypotheses (Boxes A, B) in a cross-sectional design.

## Methods

### Subjects and Data Collection

Subjects from the German-speaking part of Switzerland were referred by occupational physicians or case managers of the worker’s compensation insurance for an interdisciplinary rehabilitation assessment at the rehabilitation clinic in Bellikon (Switzerland). Subjects were insured by the Swiss Accident Insurance Fund (SUVA), the largest accident insurance in Switzerland, which covers injuries from occupational and non-occupational accidents for employed and non-employed subjects. Injured subjects receive compensation of up to 80 % of the previous salary, medical and vocational assistance up to a maximum of 2 years, and disability pensions caused by an injury.

The reason for being referred to this assessment was that subjects had not regained full working capacity within 6–12 weeks after the initial injury, had surpassed expected injury healing times, or had plateaued with medical and other rehabilitative interventions. Inclusion criteria were neck pain due to a whiplash-associated injury according the Québec Task Force (QTF) Classification of WAD, grade I (pain, stiffness, or tenderness without physical signs) or grade II (pain, stiffness, or tenderness with reduced range of motion and point tenderness), sufficient language skills to communicate with the assessors in German language and able to fill out questionnaires in German or Serbo-Croatian, Albanian, Italian, or Spanish (representing the largest immigrant groups in Switzerland) [25], aged 18–65 years, and willingness to participate. Exclusion criteria were main musculoskeletal problem not in the head and neck region, co-morbidity that considerably limited function, such as neurological deficits, rheumatoid diseases, fractures, tumours, osteoporosis, severe psychiatric disorders, pregnancy, and severe cardiac hypertension. All participants were asked for participation prior the interdisciplinary assessment. Participants were informed that they would be allowed to withdraw their participation at any time without disclosing reasons and without consequences for their medical care. The study was performed in accordance with the ethical standards of the Declaration of Helsinki and ethical approval for this study was granted by the Medical Ethics Committee of the Canton Aargau (EK AG 2010/055).

Participants’ characteristics were recorded prior to the FCE, and included age, gender, body mass index, marital status, education, native language, duration since injury, education, litigation, work capacity, education status, and physical work demands. After the determination of eligibility for inclusion in the study, patients filled out self-reported measures, i.e., questionnaires (30 min) and carried out FCE tests (20 min).

### Measurements

The WAD FCE analysed in this study consisted of tests involving activities of the upper extremities and the neck region, hand grip strength (left and right), lifting waist to overhead, overhead work, and repetitive reaching, left to right and right to left (Appendix 1). The reliability of all four FCE tests is good to excellent and the tests are safe in WAD [26]. Participants were briefly instructed on how to perform each test. The evaluator first gave a single demonstration of each test. The lifting test was commenced with a light weight. Participants were then asked to perform the test to their maximum ability. The weights lifted were incrementally increased according to a participant’s performance, using weights of 2.5 and 5 kg. To determine the level of physical effort, testers used observational criteria indicating physical demand [7]. Testing could be terminated for four reasons: the participant stopped because of, for example, pain; the observer deemed testing to have become unsafe based on biomechanical criteria; heart rate exceeded 85 % of the age-related maximum (220 minus age of the participant); or a predefined time limit was reached. If a participant stopped the lifting waist to overhead test before the criteria for maximum level of demand was observed, the highest weight in kilogram that the patient was willing to lift five times was recorded.

Pain intensity was measured with an 11-point Numeric Rating Scale (NRS) ranging from no pain (0) to worst pain (10). The patient was asked to rate his momentary pain (“pain now”), his worst and his mildest pain during the last 7 days (“maximum pain” and “minimum pain”, respectively). The NRS is a commonly used scale with proven reliability and validity in patients with neck pain [27].

The Spinal Function Sort (SFS) was used to measure self-reported functional ability to perform work-related tasks and activities of daily life that involve the spine [28]. The SFS contains 50 drawings with simple verbal descriptions of activities of material handling (e.g. lifting a 10 kg milk-crate from eye-level to the floor), postural tolerance (e.g. wash dishes at a sink) and ambulation (e.g. push and pull a shopping cart). Participants rated functional ability for each activity from “unable” (0) to “able” (4). The SFS yields a single rating ranging from 0 to 200, with higher scores indicating higher or better abilities. The scores can be categorised according the work demands as defined by the Dictionary of Occupational Titles (DOT) [29], allowing a comparison with self-reported functional abilities and work demands (sedentary to lifting weights of over 50 kg). Most patients can fill out the SFS in 10–15 min. The SFS has a good reliability and high predictive validity for non-return to work in patients with back pain [14, 30].

Neck pain-related disability was measured with the Neck Disability Index (NDI). The NDI contains 10 items: pain intensity, personal care, lifting, reading, headaches, concentration, work, driving, sleeping, and recreation. The scale of each item ranges from no disability (0) to total disability (5). The interpretation for the NDI scores is: 0–4 = none; 5–14 = mild; 15–24 = moderate; 25–34 = severe; over 35–50 = complete disability [31]. The German version of the NDI is reliable and valid [32].

The Hospital Anxiety and Depression Scale (HADS) was used to assess the symptom severity of anxiety disorders and depression in non-psychiatric populations. The HADS consists of two scales, one for anxiety and one for depression (A and D scales, respectively). Each scale contains seven items, with each item rated from 0 (best) to 3 (worst). The scale scores are calculated by summing the responses to the items up to a maximum score of 21 points (severe case) per scale. Scale scores of between 8 and 10 identify mild, 11–15 moderate, and 16 or above severe cases of anxiety/depression. Good reliability and validity, and excellent screening properties have been reported for the use of the HADS in the general population and various clinical populations [33].

### A Priori Hypotheses

#### Construct Validation: Known Groups

Four hypotheses based on known groups are displayed in Box A [11, 12]. These hypotheses were based on the fact that males are stronger than females, and, therefore, males were expected to outperform females in the strength test, but not in other tests [11].

#### Construct Validation: Hypothesis Testing

Twenty-five hypotheses on the strength of the association of FCE tests and the additional construct variables were formulated a priori. The theoretical basis for the hypotheses is explained in the introduction. Hypotheses were inferred based on previous studies with patients with chronic low back pain: it was expected that WAD FCE is correlated to a higher extent with measures of perceived ability and disability than with measures of mental distress or pain [9, 14, 34]. The strength of the association is expressed in the absolute value of the correlation coefficient. From the 25, 20 hypotheses were tested about the relationship between four FCE tests and five other construct variables (displayed in Box B). Five out of 25 hypotheses for two groups with different cultural backgrounds were formulated: four hypothesis regarding the differences of FCE test results between the two groups differed significantly and, one hypothesis was formulated that no major differences in correlations between FCE tests and construct variables exist between the two groups [effect size (ES) of the correlation coefficients <0.2]. Definitions of ES for differences between two correlations are as follow: ES ≤0.20 (small), 0.20 < ES ≤ 0.50 (medium), 0.50 < ES ≤ 0.80 (large) [35]. The two groups with different cultural backgrounds were characterized based on the native i.e. the mother language of the participants.

### Data Analysis

Normal distribution was visually assessed using P–P plots. Floor and ceiling effects were considered to be present if more than 15 % of participants achieved the lowest or highest possible score of the overhead working test [37]. The overhead working test was expected to display ceiling effects because the test was limited to a maximum of 5 min.

Associations were calculated using Pearson correlation coefficient for bivariate normally distributed data, or else a Spearman rank correlation coefficient. For relationships between gender and overhead working, and repetitive reaching, respectively, equivalence testing was performed [38]. Equivalence is established if 10 % the margins of differences between gender fall within the 90 % confidence intervals of the difference [38]. To analyse differences between genders and between two groups with different cultural backgrounds, independent sample *t* test, a Mann–Whitney U test, χ^2^ test, or linear regression was used as appropriate. The validity of the WAD FCE was considered confirmed when no ceiling or floor effects were observed in the FCE tests and the majority (80 %) of the 29 a priori hypotheses were confirmed [39]: four hypotheses concerning the relationship between FCE tests and gender, 20 hypotheses concerning the associations of the FCE tests and the other construct variables and five hypotheses concerning the two groups with different cultural backgrounds. Validity was confirmed when, significant differences in FCE test results emerged between the two groups in all 4 comparisons, and the ES for differences in correlations between FCE tests and the five construct variables between both groups was ≤0.2 in 16 or more of the 20 comparisons. The ES for differences between correlations of the two groups were calculated by subtracting the Z score of the German mother language group by the Z score of the non-German mother language group. Z scores were calculated as follows: 0.5 ln [(1 + r)/(1 − r)], were r is the correlation coefficient between an FCE test and a reference measure [35]. *p* < 0.05 was used as a cut-off, indicating statistical significance. For readability, the terms confirmed/not confirmed were used instead of not rejected/rejected to indicate the interpretation of the results concerning the hypotheses. Methodologically, the terms not rejected/rejected are more correct. All analyses were performed using SPSS (Statistical Package for Social Sciences, Version 21, IBM Corp.).

## Results

### Participants

From January 2011 to January 2012, 428 patients were referred for interdisciplinary assessment due to delayed recovery after musculoskeletal injury. From the referred patients (n = 114), 79 (69 %) were not eligible because the main problem was not in the neck and head region; 17 (15 %) had insufficient German language skills to communicate with the assessors or not able to fill out the questionnaires in the language versions available; 5 (5 %) had acute comorbidity that limited testing, such as fracture or severe psychiatric disorder; 2 (2 %) were pregnant; 6 (5 %) were excluded due to other medical reasons; 3 (3 %) due to age under 18 or over 65 years; and 2 (2 %) were of grade III–IV by QTF criteria.

In total, 314 patients fulfilled the inclusion criteria and participated in this study. The participants’ characteristics are presented in Table 1. Participants’ characteristics were analysed in two groups with cultural differences, n = 152 (48 %) participants with German as their native language and n = 162 (52 %) with a non-German language as their native language. Significant differences between the groups were observed in 8 out of 10 main participant characteristics (Table 1). In five self-reported measures (Table 1), significant differences were found between the two groups.

**Table 1:** Characteristics of the participants

Characteristics, unit or scale	Total n = 314	German n = 152	Non-German^a^ n = 162	*p* value^h^
Age (years), median (IQR)^b^	36.0 (27.0–45.0)	34.5 (26.0–46.0)	36.0 (29.9–44.3)	<0.476^i^
Gender female, n (%)	133 (42.4)	83 (54.6)	50 (30.9)	<0.001^k^
BMI^d^, median (IQR)^b^	26.0 (22.0–30.0)	24.0 (21.0–29.0)	27.0 (24.0–30.0)	<.001^i^
Marital status, n (%)				
Married or co-habitation	161 (51.3)	40 (26.3)	121 (74.1)	<0.001^j^
Single	109 (34.7)	85 (55.9)	24 (14.8)	
Divorced or living separated	42 (13.4)	26 (17.1)	16 (9.9)	
Other	2 (0.6)	1 (0.7)	1 (0.6)	
Duration since WAD injury claim opening (days), median (IQR)	91.0 (72–124.0)	91.0 (72.0–122.5)	91.0 (73.5–126.3)	<0.986^i^
Attorney involved, n (%)	86 (27.4)	37 (24.3)	49 (30.2)	<0.025^j^
Work incapacity in % previous work^e^, median (IQR)	80 (40–100)	50 (25–100)	100 (50–100)	<0.001^i^
Education^f^, n (%)				
low	147 (46.8)	33 (21.8)	114 (70.4)	<0.001^j^
intermediate	159 (50.6)	113 (74.3)	46 (28.4)	
high	8 (2.5)	6 (3.9)	2 (1.2)	
Physical work demands^g^ n (%)				
sedentary to light (<5–10 kg)	110 (35.0)	74 (48.7)	36 (22.2)	<0.001^j^
light to medium (11–25 kg)	113 (36.0)	42 (27.7)	71 (43.8)	
eavy to very heavy (26 to >45 kg)	91 (29.0)	36 (23.6)	55 (34.0)	
Pain intensity (NRS 0–10) Mean (SD)				
Pain now mean (SD)	4.6 (2.2)	4.2 (2.3)	4.9 (2.2)	<0.002^l^
Pain maximum, last 7 days, median (IQR)^b^	8.0 (6.0–9.0)	7.5 (5.3–8.0)	8.0 (6.8–9.0)	<0.011^i^
Pain minimum, last 7 days, median (IQR)^b^	3.0 (1.0–4.0)	2.0 (0.0–3.0)	3.0 (2.0–5.0)	<0.001^i^
Perceived functional ability (SFS 0–200), median (IQR)^b,c^	141.0 (103–163)	151.7 (128–174)	120.0 (91–158)	<0.001^i^
Disability (NDI 0–50), mean (SD)	22.5 (8.3)	20.9 (7.9)	24.0 (8.3)	<0.001^l^
Anxiety (HADS 0–21), median (IQR)^b^	9.0 (5.0–12.0)	6.0 (4.0–10.0)	11.0 (7.0–14.0)	<0.001^i^
Depression (HADS 0–21), median (IQR)^b^	7.0 (3.8–10.0)	5.0 (2.0–8.0)	8.5 (5.8–12.00)	<0.001^i^

*NRS* Numeric Rating Scale; *NDI*  Neck Disability Index, *HADS* Hospital Anxiety and Depression Scale, *SFS* Spinal Function Sort

^a^Native language: Albanian n = 82 (62.1 %), Serbo-Croatian n = 25 (8 %), Italian = 17 (5.5 %), Other n = 28 (8.8 %; Turkish, Arabic, Portuguese, Spanish). ^b^ Data with a skewed distribution are presented with a median and an interquartile range (IQR). ^c^ Data missing for 7 participants ^d^ *BMI* body mass index, ^e^ work incapacity set by the insurance assessed for the actual or previous job (if jobless) in % at the time of WAD FCE, ^f^ low = no vocational education, intermediate = vocational education, high = bachelor or higher education, ^g^ Maximum physical work load of material handling tasks according to the Dictionary of Occupational Titles (DOT). Category light to medium was added to ensure that all participants could be categorized in a continuous scale. ^h^ *p* value = significant, if *p* < 0.05 concerning differences between men and female based on the results of ^i^ Mann–Whitney U test, ^j^ skewed distribution of scaled data, ^k^ χ^2^ test for categorical data, and ^l^ *t* test for continuous data

### Descriptive Analysis of FCE Test Results

Normal distribution was found in three out of four FCE tests, i.e., lifting waist to overhead, hand grip strength (right), and repetitive reaching (right). A ceiling effect was observed in the overhead working test with 38 % (n = 119) of the participants reaching the maximum time limit of 300 s. Between the two language groups and genders, the differences in FCE tests were significant in six out of eight comparisons (Table 2). There was no significant interaction between gender and language.

**Table 2:** Differences in FCE results between language groups and gender

FCE tests (unit), Mean (SD)	German		Non-German		*p* value*	
	Males n = 69	Females n = 83	Males n = 112	Females n = 50^a^	Gender differences	Language differences
Hand grip strength right (kgF)	45.9 (12.1)	26.0 (8.1)	37.3(12.9)	18.4 (8.2)	<0.001	<0.001
Lifting waist to overhead (kg)	14.8 (6.4)	10.3 (4.0)	11.9 (6.0)	7.3 (3.7)	<0.001	<0.001
Overhead working (s)	228.2 (90.0)	222.3 (94.9)	157.8 (95.9)	141.4 (92.0)	0.322	<0.001
Repetitive reaching right (s)^a^	76.9 (20.3)	70.7 (25.2)	88.4 (28.1)	84.63 (28.8)	0.098	<0.001

*SD* standard deviation

* Based on the results of a linear regression analysis

^a^Data missing for one participant

### Construct Validation: Known Groups

As presented in Table 3, men had a significantly greater hand grip strength (+17.5 kg), and lifted significantly more weight over head (+3.7 kg). Differences between genders were in the overhead working test −7.4 s and the repetitive reaching test −8.2 s. The 10 % margin of differences between gender for overhead working was 18.5 s (90 %CI −26.2 to 11.4) and for repetitive reaching 8.8 s (90 %CI 3.2–13.2). The 90 % CI did not fall within the 10 % margin, thus non equivalence could not be ruled out. Two out of four gender-related hypotheses were confirmed.

**Table 3:** Differences in FCE tests results between genders

FCE tests (unit)	Males n = 181		Females n = 133		*p* value^a^
	Mean	SD	Mean	SD	
Hand grip strength right (kgF)	40.6	13.3	23.1	8.9	<0.001
Lifting waist to overhead (kg)	13.0	6.3	9.2	4.1	<0.001
Overhead working (s)	184.6	99.4	192.0	101.4	0.557^#^
Repetitive reaching right (s)	84.0	26.0	75.8	27.3	<0.001^#^

“ceiling effect” at 300 s

*SD* standard deviation

^a^ *p* value = significant, if *p* < 0.05; ^#^ Mann–Whitney U test

### Construct Validation: Hypothesis Testing

Correlations between the FCE tests and pain, perceived functional ability, disability, anxiety, and depression are presented in Table 4. For each of the FCE tests, four out of five hypotheses were confirmed.

**Table 4:** Correlations between the results of FCE tests and pain, perceived functional ability, disability, anxiety, and depression to test construct validity of FCE tests, for the total group

FCE tests	Pain now (NRS)	Functional ability (SFS)	Disability (NDI)	Anxiety (HADS A)	Depression (HADS D)
Hand grip strength right (kgF) 95 % CI	−0.26 (−0.36 to −0.16)	0.38 (0.28 to 0.47)	−0.26 (−0.36 to −0.15)	−0.28 (−0.38 to −0.17)	−0.25 (−0.35 to −0.15)
Lifting waist to overhead (kg) 95 % CI	−0.39 (−0.48 to −0.29)	0.60 (0.52 to 0.66)	−0.39 (−0.48 to −0.29)	−0.27 (−0.37 to −0.16)	−0.30 (−0.40 to −0.20)
Overhead working (sec) 95 % CI	−0.36 (−0.46 to −0.26)	0.61 (0.54 to 0.68)	−0.45 (−0.53 to −0.35)	−0.36 (−0.45 to − 0.26)	−0.41 (−0.50 to −0.31)
Repetitive reaching right (sec) 95 % CI	0.31 (0.20 to 0.40)	−0.42 (−0.50 to −0.32)	0.34 (0.23 to 0.43)	0.27 (0.16 to 0.37)	0.34 (0.24 to 0.43)

The Pearson correlation statistic was used. All correlations were significant at the *p* value 0.01 level (2-tailed). *CI* Confidence interval. Interpretation: *NRS* Numeric Rating Scale, *SFS* Spinal Function Sort, *NDI* Neck Disability Index, *HADS A* Hospital Anxiety and Depression Scale, subscale Anxiety, *HADS D* Hospital Anxiety and Depression Scale, subscale Depression

Correlations for the two language groups between the four FCE tests and the reference measures are presented in Table 5. Eighteen out of 20 ES were ≤0.20 (ranging from 0.01 to 0.16). In two comparisons, the ES for the difference in correlations between groups with different cultural backgrounds was >0.20; −0.21 for lifting waist to overhead and the SFS, and 0.22 for lifting waist to overhead and HADS anxiety (ES data available from the author on request). The hypothesis on the validity of FCE tests in patients with cultural differences was confirmed because ES were ≤0.20 in the 18 of 20 comparisons.

**Table 5:** Correlations between the results of FCE tests and pain, perceived functional ability, disability, anxiety, and depression separated by language groups

FCE tests	Pain now (NRS 0–10)		Functional ability (SFS 0–200)		Disability (NDI 0–50)		Anxiety (HADS A 0–21)		Depression (HADS D 0–21)	
	German	N-German	German	N-German	German	N-German	German	N-German	German	N-German
Hand grip strength right (kgF)^a^ 95 % CI	−0.24* −0.38 to −0.08	−0.26 −0.40 to −0.11	*0.26* *0.10–0.40*	*0.43* *0.30–0.55*	−0.16* −0.31 to 0.00	−0.32 −0.45 to −0.17	−0.24 −0.38 to −0.08	−0.27 −0.40 to −0.12	−0.18* −0.33 to −0.22	−0.27 −0.40 to −0.12
Lifting waist to overhead (kg)^a^ 95 % CI	−0.41 −0.53 to −0.26	−0.33 −0.46 to −0.19	*0.50* ^*c*^ *0.37*–*0.61*	*0.64* ^*c*^ *0.54*–*0.73*	−0.34 −0.47 to −0.19	−0.40 −0.52 to −0.26	−*0.12* ^*†d*^ −*0.27 to 0.04*	−*0.32* ^*d*^ −*0.45 to* −*0.18*	−0.19* −0.34 to −0.03	−0.32 −0.45 to −0.18
Overhead working (s)^b^ 95 % CI	−0.39 −0.52 to −0.25	−0.28 −0.41 to −0.13	0.60 0.48–0.69	0.52 0.40–0.63	−0.42 −0.54 to −0.28	−0.40 −0.52 to −0.26	−0.20* −0.35 to −0.04	−0.30 −0.43 to −0.15	−0.26 −0.41 to −0.11	−0.35 −0.48 to −0.20
Repetitive reaching right (s)^b^ 95 % CI	0.28 0.13–0.42	0.27 0.12–0.41	−0.42 −0.54 to −0.28	−0.36* −0.49 to −0.21	0.39 0.25–0.52	0.29 0.14–0.43	0.17* −0.41 to −0.11	0.19* 0.04–0.34	0.30 0.14–0.44	0.26 0.11–0.40

*NRS* Numeric Rating Scale, *SFS* Spinal Function Sort, *NDI* Neck Disability Index, *HADS A* Hospital Anxiety and Depression Scale, subscale Anxiety, *HADS D* Hospital Anxiety and Depression Scale, subscale Depression, *CI* confidence interval

^a^ Pearson correlation statistic, ^b^ Spearman’s rank correlation statistic. All values presented were significant at *p* < 0.01 (2−tailed), except * *p* < 0.05 (2-tailed), ^†^ *p* = 0.15. Non-italics: difference in correlation between cultural groups ES ≤0.20. Italics: difference ES >0.20: ^c^ ES = 0.214, ^d^ ES = 0.215

To summarize, from a total of 29 a priori hypotheses, 23 (79 %) were confirmed (for an overview see Appendix 2).

## Discussion

The aim of the study was to analyse construct validity of FCE tests for application in patients on workers’ compensation due to WAD grade I and II across groups with cultural differences (defined as the native language of the participant). Twenty-three out of 29 (79 %) instead of the expected 80 % of the a priori defined hypotheses were confirmed. Confirmed were 2 out of 4 gender-related hypotheses, 5 out of 5 culture-related hypotheses, and 16 out of 20 construct-related hypotheses (overview in Appendix 2). Differences in correlations between the groups with cultural differences were statistically significant, but small (18 out of 20 ES were ≤0.2) despite large differences in patient characteristics and FCE performances. A ceiling effect was observed in 1 test (overhead working). Overall, the construct validity was confirmed for the majority of FCE tests for testing functional capacity in patients with WAD with cultural differences and in a workers’ compensation setting.

The results of the study support the bio-psycho-social construct of FCE in WAD: we observed differences between males and females (bio), between language groups (socio), and small but consistent relationships with psychological factors (psycho). The gender differences in FCE tests in this study are consistent with the results of others [11]. Differences in test results, but not in correlations, were observed between language groups. The non-German language group consisted of individuals from the largest immigrant groups in Switzerland [25]. The participants of this study consisted of 52 % whose native language was non-German, which is higher than the 18 % of the Swiss population [25]. The proportion of male participants in the non-German group in this study was similar (47.6 %) to that of the Swiss working population (51 %) [25], but higher than usually reported in WAD [1]. These differences may be explained by the fact the study participants were insured by SUVA, which insures many companies from the industry and construction sector, where the rate of male, non-German speaking subjects is higher than in the other business sectors [40]. Many immigrants have been naturalised to Swiss citizenship, hence native language was chosen as an indicator for cultural differences. Native language has been reported as a valid indicator for cultural differences [41]. A study on the coping styles of patients with low back pain found large differences among groups with different native languages in Switzerland [42].

To test construct validity, associations were made with other constructs known to be associated with FCE outcomes. In two out of four instances, the associations between gender and FCE outcomes occurred as hypothesized. Although differences were small in the overhead working and repetitive working tests, equivalence between genders could not be ruled out. We expected no difference between genders, because for this test muscle force is not likely primary factor for outcome. In the healthy population, conflicting evidence for the difference between genders in dexterity performance tests has been reported [12, 43, 44]. Results in fine manual dexterity tests may be influenced by finger size; smaller fingers were related to better outcomes [45]. This might be a plausible explanation of the results of this study.

In patients with CLBP, moderate correlations between FCE and SFS [14], and between FCE and other self-reported measures of disability were reported [9]. In this study, FCE correlated more strongly with SFS (moderate correlations) than with the NDI (weak correlations). There could be several explanations for this. Firstly, the items of the SFS more closely resemble the items of the FCE than the NDI. Secondly, inconsistent wording of the NDI items concerning the influence of pain on activity levels may partly explain the results. Thirdly, while our hypothesis was based on the majority of the studies in CLBP where the relationship between FCE and self-reported disability was moderate, this relationship may be slightly different in patients with WAD or when using the NDI. Additionally, there may have been unknown sample characteristics contributing to these differences.

The strengths of the correlations between FCE and psychological variables in patients with WAD appear higher compared with CLBP patients [9]. This may be consistent with the relevance of psychological factors in WAD [3, 46]. We compared our results with a recently published study with 40 patients with WAD from the Netherlands [47]. On average the Dutch sample was younger (mean 33 years, SD 9.6), more female (55 %) and the duration since whiplash injury was longer (median 12 months, IQR 7–19). While the results of the repetitive reaching test between the two samples were similar (mean difference 2 s), the differences between the lifted weight from waist to overhead between the Dutch and the Swiss patients with WAD was substantial (the Dutch lifted a mean of 12.2 kg more). The differences between the studies might be explained by sample variation since sample in the Dutch study was small. But these differences need further investigation. Nevertheless, they are consistent with a study that reported large differences in FCE outcomes between different countries in patients with low back pain [19]. The strength of the correlations between NDI and lifting waist to overhead and overhead working between the Dutch and the Swiss WAD samples were similar, suggesting some robustness of the results between study samples from different countries. Shortly, these findings underline the importance of replication of validation studies among different (social security) contexts.

Some potential limitations have to be addressed. The study population consisted of injured workers who did not return to work within the first 6–12 weeks, for whom recovery had plateaued, and who were referred by the case manager or occupational physician. The validity of WAD FCE should also be established in other WAD patients outside the workers’ compensation setting, in general practice or in more chronic WAD patients (in rehabilitation settings). Moreover, the a priori defined hypotheses were based on previous studies performed in populations other than WAD. Most studies reported conflicting evidence on many FCE-related factors [9], so cut-offs for the strength of the correlation were arbitrarily chosen. Additionally, if other measures for construct validation had been used, the results might have been different. In this study, self-reported measures were used, which are related to physical capacity but distinct [48–50].

In the overhead working test, a ceiling effect was found in 38 % of the participants, as reported for healthy subjects and CLBP patients [51, 52]. It was not expected that such a high proportion of patients with WAD would reach the time limit of 300 s, because one could suppose a reduced postural tolerance in the neck and upper limbs. For future research, we suggest modifying the overhead working test by having the subject wear two cuff weights of 1 kg each around on their forearm to reduce ceiling effects, as described for healthy subjects [53].

The strengths of this validation study of FCE for WAD patients were the use of a priori defined hypotheses in the analyses, allowing transparency and explicitness. Therefore, several comparisons could be made to a variety of constructs, enabling the reader to interpret the validity from different points of views. Additionally, the design and the sample size of the current study meet the proposed quality standards for FCE validation studies [22]. Moreover, patients with different cultural backgrounds participated in our study, unless previous FCE studies where languages or cultural differences were not reported [9]. To our knowledge, this has not been the subject of a study in a setting similar to ours (validation of FCE tests). Although replication is needed, the results of this study support the validity of the WAD FCE in patients with different native languages (i.e., cultural backgrounds).

## Conclusion

The construct validity was confirmed for the majority of FCE tests for testing functional capacity in patients with WAD with cultural differences and in a workers’ compensation setting. Additional validation studies in other settings are needed for verification.

## Appendix 1: Materials and Procedures of the WAD FCE

### Isometric Hand Grip Strength

Isometric hand grip strength was measured in a seated position. The subjects held their shoulder adducted without internal or external rotation, elbow flexed at approximately 90° and the forearm and wrist in neutral position. Grip strength of the right hand was measured in a three-trial procedure while maintaining in a hand dynamometer in one single handgrip position adapted to the handsize of the subject (Jamar PC 5030, Preston Corporation, 1994). An average amount of kilogram-force was scored.

### Lifting Waist to Overhead Test

Lifting waist to overhead was measured during 5 lifts of the crate from table to crown in standing position, and vice versa within 90 s in standing position. The test was executed with a wooden crate (40 × 30 × 26 cm) of 2.5 kg. Weight increments of 2.5 or 5 kg each were used until the maximum amount of weight was reached. Maximum performance was recorded in kg.

### Overhead Work Test

Overhead working was performed standing with hands at crown height for manipulation of nuts and bolts. The ceiling of the test was 5 min. The time that the position was held was recorded (s).

### Repetitive Reaching Test

Repetitive reaching was determined by fast horizontal movements of the upper extremity in a sitting position. Marbles were removed from bowls at arm length distance at table height from left to right and vice versa, with the right arm. The time taken to remove 30 marbles was recorded (s).

## Appendix 2

See Table 6.

**Table 6:** Overview of all a priori hypotheses (n = 29) and interpretation of results

n = of hypotheses	Type of construct validity	Reference test	Construct validity is confirmed when mean performance:	r cut-off values for confirmed hypotheses	Interpretation of results^a^ (n of confirmed hypotheses)
1	Gender differences	Lifting waist to overhead (kg)	Females < males	difference ≥ 10 %; *p* ≤ 0.05	Confirmed (1)
1	Gender differences	Isometric hand grip strength right (kgF)	Females < males	difference ≥ 10 %; *p* ≤ 0.05	Confirmed (1)
1	Gender differences	Overhead working (s)	Females ≈ males	difference < 10 %; *p* > 0.05	Not confirmed^b^ (0)
1	Gender differences	Repetitive reaching right (s)	Females ≈ males	difference < 10 %; *p* > 0.05	Not confirmed^b^ (0)
			Construct validity is confirmed when the strength of the relationship of four FCE tests^a^ with		
4	4 FCE tests and construct variables	Pain now (NRS)	Pain is low or weak	0.25 < |r| < 0.50	Confirmed (4)
4	4 FCE tests and construct variables	Functional ability (SFS)	Self-reported functional ability is low to moderate	0.25 < |r| ≤ 0.70	Confirmed (4)
4	4 FCE tests and construct variables	Disability (NDI)	Self-reported disability is moderate	0.50 ≤ |r| ≤ 0.70	Not confirmed (0)
4	4 FCE tests and construct variables	Anxiety (HADS A)	Anxiety is low or weak	0.25 < |r| < 0.50	Confirmed (4)
4	4 FCE tests and construct variables	Depression (HADS D)	Depression is low or weak	0.25 < |r| < 0.50	Confirmed (4)
4	4 FCE tests and 2 groups with different cultural background	German speaking vs Non-German speaking Group	Construct validity is confirmed when FCE test results differ significantly between groups with different cultural background	p < 0.05	Confirmed (4)
1	Strength of associations between 4 FCE tests and construct variables for two groups with different cultural background	Correlation coefficients between for FCE tests and NRS, SFS, NDI, HADS A and HADS D	Construct validity is confirmed when the majority of associations in difference of strength of the relationship between the two cultural groups for four FCE tests with the construct variables NRS, SFS, NDI, HADS A and HADS D have a small effect size^c^	ES ≤ 0.20 (small)	Confirmed (1)
Total confirmed					23

|r| = correlation coefficient, absolute value, *ES* effect size, *NRS* Numeric Rating Scale, *SFS* Spinal Function Sort, *NDI*  Neck Disability Index, *HADS A* Hospital Anxiety and Depression Scale, subscale Anxiety, *HADS D* Hospital Anxiety and Depression Scale, subscale Depression

^a^Hypotheses confirmed (= not rejected); hypotheses not confirmed (=rejected); ^b^ hypotheses not confirmed, based on results of equivalence testing; ^c^ “small” = ES ≤0.20

**Box A:** A priori hypotheses about the relationship between FCE tests and gender

FCE test	Construct validity is confirmed when mean performance:
Lifting waist to overhead (kg)	Females < males (difference ≥ 10 %; *p* ≤ 0.05)
Isometric hand grip strength right (kgF)	Females < males (difference ≥ 10 %; *p* ≤ 0.05)
Overhead working (s)	Females ≈ males (difference < 10 %; *p* > 0.05)
Repetitive reaching right (s)	Females ≈ males (difference < 10 %; *p* > 0.05)

**Box B:** A priori hypotheses about the relationship between 4 FCE tests^a^ and 5 construct variables

Reference test	Construct validity is confirmed when the strength of the relationship of four FCE tests^a^ with	r cut-off values
Pain now (NRS)	Pain is low or weak	0.25 < |r| < 0.50
Self-reported functional ability (SFS)	Self-reported functional ability is low to moderate	0.25 < |r| ≤ 0.70
Self-reported disability (NDI)	Self-reported disability is moderate	0.50 ≤ |r| ≤ 0.70
Anxiety (HADS A)	Anxiety is low or weak	0.25 < |r| < 0.50
Depression (HADS D)	Depression is low or weak	0.25 < |r| < 0.50

^a^FCE includes the tests Lifting waist to overhead (kg), Hand grip strength right, (kgF), Overhead working (s), Repetitive reaching right (s); |r| = correlation coefficient, absolute value. The direction of the association depends on the scoring of the reference measure. Interpretation: 0.00–0.25 little if any (“not correlated”); 0.26–0.49 low or weak; 0.50–0.69 moderate; 0.70–0.89 high or strong; 0.90–1.00 very strong correlation [36]
